# Effect of Task-Specific Training on Trunk Control and Balance in Patients with Subacute Stroke

**DOI:** 10.1155/2020/5090193

**Published:** 2020-11-17

**Authors:** Mohamed E. Khallaf

**Affiliations:** ^1^Department of Physical Therapy for Neuromuscular Disorders and Its Surgery, Faculty of Physical Therapy, Cairo University, Giza, Egypt; ^2^University of St. Augustine for Health Sciences, Austin, Texas, USA

## Abstract

**Objectives:**

Impairment of static and dynamic posture control is common after stroke. It is found to be a predictor and an essential component for balance, walking ability, and activities of daily living (ADL) outcomes. Studies investigating effect of physical therapy techniques with an aim to improve trunk function after stroke are limited. This study aimed at studying the effect of task-specific training on trunk control and balance in patients with subacute stroke.

**Methods:**

In this randomized controlled trail, thirty-four patients were alienated into two equal groups. The study group (*n* = 17) received task-specific training, and the control group (*n* = 17) received conventional physical therapy based on the neurodevelopmental technique. Task-specific training was applied through two phases with criteria of progression based on Chedoke–McMaster Stroke Assessment postural control stages. The interventions were applied in a dosage of 60 min per session, three times a week for ten weeks. Static and dynamic balance were measured by the trunk impairment scale (TIS), postural assessment scale (PAS), and functional reach test (FRT). Laser-guided digital goniometer was used to measure the trunk ranges of motions (ROM) as a secondary outcome.

**Results:**

Significant differences between the baseline and the follow-up measures including TIS, PAS, FRT, and trunk (ROM) were found in both groups (*P* ≤ 0.05). In-between group comparison also showed significant differences between the results of both groups indicating more improvements among patients representing the study group.

**Conclusion:**

Task-specific training may be effective in improving the static and dynamic postural control and trunk ranges of motion among subacute stroke patients.

## 1. Introduction

Impaired trunk control is common among stroke survivors [1]. Existing studies have reported many causes including muscle weakness, spasticity, delayed activity of the trunk muscles, perceptual deficits [2], significant error of trunk position sense [3], inadequate center of pressure control when sitting [4], decreased trunk performance [5], and trunk asymmetry during gait [6]. Moreover, anticipatory control might be disturbed in stroke patients [7]. These impairments are directly related to an increased risk of fall, impaired mobility, and participation restrictions [8]. This also could lead to high levels of disability and dependency in patients' activities of daily living [8, 9].

After stroke, trunk function is found to be a predictor and an essential component for respiratory functions, balance, walking ability, and activities of daily living (ADL) outcomes [10–13]. So, a significant relation between bilateral trunk muscle performance (encompassing strength, power, and muscular endurance) and measures of balance, gait, and functional ability after stroke has been proved. Loss of trunk ability and paralysis of respiratory muscles decrease the lung and chest expansion which may progress to complications such as pneumonia [10]. Moreover, trunk performance assessed at the admission to a rehabilitation center was the most important predictor of functional recovery at the time of discharge six months after stroke [14]. On admission, the static sitting balance is the most important factor in predicting performance of many ADL including feeding, transfer, personal care, toilet use, bathing, dressing, and bowel and bladder control. The primary contribution of the trunk muscles is to stabilize the spine and trunk. This stabilization is conditional for free and selective movements of the head or extremities [15].

Despite evidence showing how important is the trunk control as a prognostic factor for decreasing activity limitations and improving participation after stroke, there is a dearth of studies on trunk control in stroke patients' rehabilitation and often conducted less than studies on upper and lower extremities. Studies investigating the effect of therapy with an aim to improve trunk function are limited, and the results are contradicting. The effectiveness of three different therapy approaches (neurodevelopmental technique (NDT), specific reaching tasks, and balance training) was compared, and the NDT method was reported to be the most effective for trunk development [16]. However, Pollock et al., reported that trunk training with the NDT has no effect on sitting balance [17]. An audiovisual biofeedback-based trunk stabilization training was used in training of stroke patients with impaired sitting balance. A significant improvement in sitting balance was found after training for 6 weeks when compared to another group of patients who received conventional therapy only [18]. Additionally, practicing reaching tasks beyond arm's length on sitting and reaching while standing up in the chronic phase after stroke were reported to have positive effect on the trunk control [19]. It was also reported that participants who practiced task-related balance training showed a significantly larger maximum reach distance and peak vertical force through the affected foot during standing [20].

Task-specific training is a repetitive training of functional task or an element of a single functional task that is carried out in an open environment to acquire efficient and effective motor skills. The task-specific training is based on the dynamic systems theory: movement behavior is the result of complex interactions between many different subsystems in the body, the task at hand, and the environment [21]. During task-specific training, many types of movement are practiced for limiting the stereotyped movements and increasing adaptive movements [22]. With the task-specific training, the focus of rehabilitation is to enhancing function across all performance domains by emphasizing function, participation, and quality of life [23].

This study is aimed at investigating the effect of task-specific training on trunk control in patients with stroke. The novelty of this study lies in the focus on the development of an intervention protocol based on the principles of motor learning which would benefit patients who had experienced stroke. In addition, we used specific, functional, and reliable progression parameters of Chedoke–McMaster Stroke Assessment (CMSA) postural control stages [24] to transfer the performer between phases of therapy.

## 2. Subjects and Methods

**Figure 1:**
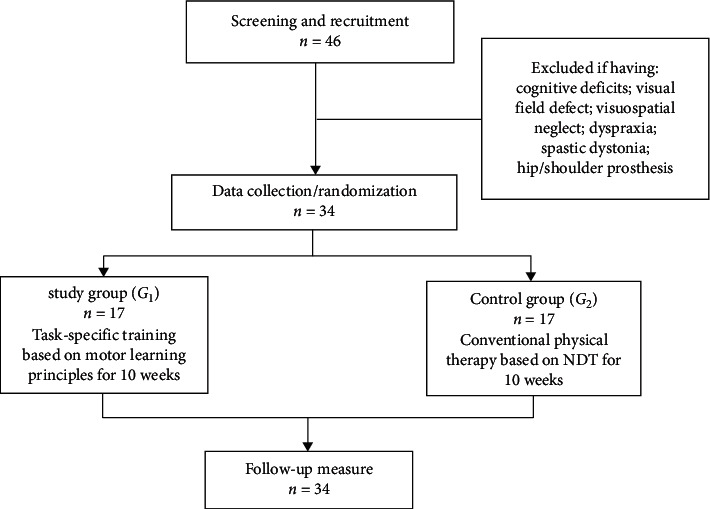
Study flowchart: *n* number; NDT: neurodevelopmental technique.

Forty-six patients with diagnosis of first-ever subacute stroke, which resulted in hemiparesis, hemihyposthesia, and impaired proprioception, were recruited from the outpatient clinic of the Faculty of Physical Therapy, Cairo University. Diagnosis was confirmed based on CT or MRI findings, and the patients were referred to the clinic by a physician. Thirty-four patients (14 females and 20 males) were eligible to participate in the study based on the following criteria: able to give informed consent; medically stable; sit without holding on (stage 2 postural control of CMSA); stand independently for at least one minute; and be able to flex the nonparetic shoulder to at least 90 degrees without holding on to be able to facilitate static righting. Patients were excluded when they did not meet the inclusion criteria or having one of the following: cognitive deficits (assessed by the Montreal cognitive assessment tool); visual field defect; visuospatial neglect; motor planning deficits (e.g., dyspraxia); affected upper/lower extremity moderate spasticity or spastic dystonia; lost superficial/deep sensations; hip prosthesis; or any other condition interfering with trunk movements other than stroke. Three patients were eligible but refused to participate because of the commute or work schedule. Participants were equally assigned into two equal groups: task-specific training group (*G*_1_; *n* = 17) and conventional training group (*G*_2_; *n* = 17). For allocation in groups, each participant blindly pulled up a sealed envelope indicating one of the treatment groups. Moreover, the envelopes were not revealed to the investigator who was responsible for the process of the envelop selection (Figure 1). The study was approved by the Institutional Ethics Committee at Faculty of Physical Therapy, Cairo University, and written informed consent was obtained from each patient.

After initial data collection, the trunk impairment scale (TIS) was used to assess the static and dynamic sitting balance and trunk coordination in a sitting position [25]. The TIS consists of three subscales: static sitting balance, dynamic sitting balance, and coordination. Each subscale contains between three and ten items. The TIS score ranges from a minimum of 0 to a maximum of 23. Postural assessment scale (PASS) was also used to assess postural control and participants' ability to maintain stable posture and equilibrium during changing positions [26]. PASS consists of a four-point scale where the items are scored from zero to three, and total scoring ranges from 0 to 36. Functional reach test (FRT) was also used to assess the anticipatory balance. The FRT was measured by asking the participant to reach forward with the nonparetic arm as far as possible without taking a step [27].

**Figure 2:**
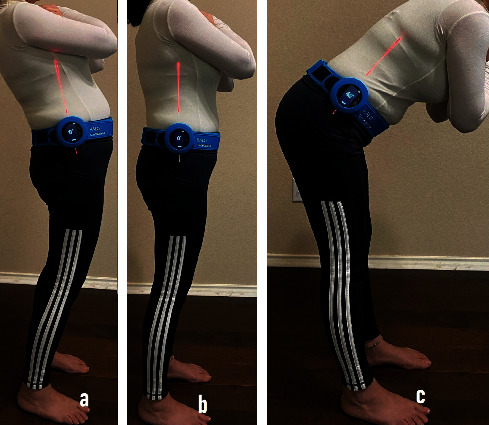
Measurement of the (a) trunk extension, (b) neutral, and (c) trunk flexion. A laser-guided digital goniometer (HALO) is fastened to a belt around the waist while standing.

**Figure 3:**
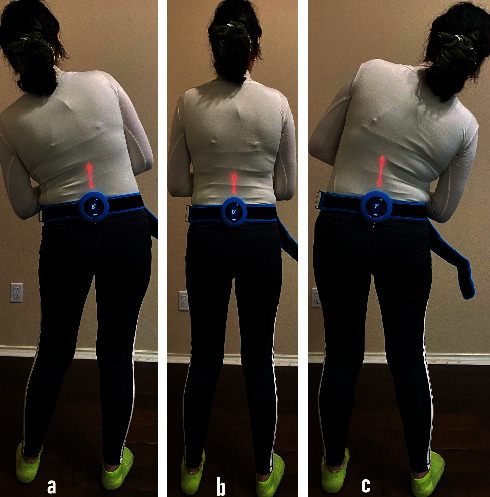
Measurement of the trunk (a) lateral flexion to the left, (b) neutral, and (c) lateral flexion to the right. A laser-guided digital goniometer (HALO) is fastened to a belt around the waist while standing.

A laser-guided digital goniometer was used to measure the flexion, extension (Figure 2), lateral flexion (Figure 3), and rotation to the right and left of the trunk (HALO, model HG1, HALO Medical Devices, Australia). It utilizes magnetic system, accelerometers, and laser that intersect with anatomical landmarks, distal and proximal to the segment being measured which reduces the need for the visual estimation. The HALO is a hand-held, pocket-sized (88 mm × 88 mm × 17 mm), digital goniometer using low-level class 1 laser technology to measure joint angles in degrees. Correll et al. provided evidence that the HALO digital goniometer can be a reliable and valid tool for measuring range of motions (ROM) with intrarater reliability between 0.82 and 0.91 and interrater reliability of 0.89 to 0.98 [28].

All patients included in *G*_1_ and *G*_2_ received a 15 min one-time lecture about the importance of trunk control for performance of daily activities as a trial to empower patients to advocate for their own health. Patients in *G*_2_ received 60 minutes three times a week over ten weeks of conventional physical therapy management for the trunk based on the neurodevelopment therapy. Treatment included facilitation of upper, lower trunk, and pelvis movements and alignment using key points of control, weight shifting in sitting, bridging, and modified bridging exercises. Additionally, strategies to improve postural control in quadruped, kneeling, half-kneeling, and standing were utilized. Changing base of support, changing supporting surface, use of upper extremity movements, and external perturbations were used as a challenge for postural control.

Participants in *G*_1_ received task-specific training where the intended movements were reinforced to be done correctly through terminal, sensory-augmented (delivery of additional task-related sensory cues, e.g., via clear and simple auditory, tactile, or visual modalities) feedback that conveys pertinent information about body orientation for balance with caution not to overload the patient with excessive or wordy commands. Knowledge of results and quality of the movement were given during execution of the task at phase I while knowledge of performance was given at phase II. As initial practice progresses, the patients were asked to self-examine performance and identify problems. Specifically, what difficulties exist? What could be done to correct the difficulties? and what movements could be eliminated or refined? Practice of incorrect movement patterns was not allowed to prevent faulty habits and postures and to enhance learning the correct pattern of movements. To improve participants' capabilities, intense, structured, and variable practice was adopted. The participants were given the opportunity to practice and practice in a closed, tightly controlled, clinic environment and then in an open constantly changing and unpredictable environment.

The exercise program was accomplished through two phases: phase I started after randomization with an aim to progress the participants from stage 2 to stage 4 postural control of CMSA. Trunk control exercises started with assistance in sitting on a treatment table with upper extremities (UEs) crossed in front of the chest, and the affected UE was lifted by the nonparetic UE or resting them on a movable table in front of the patient to avoid any stress on the glenohumeral joint. The exercise protocol included leaning to and from the affected side, moving in the sagittal and oblique planes. Moving the shoulders forward to a target; spinal rotation to the affected and then nonaffected side; flexing the hips in sitting and sliding arms forward on a table to a target; reaching to the affected side slowly; sitting on a high table with weight bearing through the affected leg; pushing down through the leg when leaning forwards; and independent rising from sitting to standing were also practiced. During this phase, physical assistance through distributed practice was utilized because of the complexity of the tasks and to enrich the learning process without the interfering effect of fatigue and other upper motor neuron lesion sequelae. We started with a sequence of practice and rest periods in which the practice time was equal to the time at rest, and gradually the training time was increased.

Once the patient was able to perform tasks of stage 4, phase II was started with an aim to reach stage 6 postural control of CMSA. Phase II included the following exercises: sitting and reaching up and down; sitting and reaching to the affected side; reaching from side to side; forward and backward in sitting; picking an object off the floor; reaching to the side when standing up; standing and looking behind (dual tasking); standing with one leg forward and looking behind (dual tasking); standing with narrow base of support; tandem and semitandem stance with and without head turns; standing on one leg with support; marching on the spot; and standing and bouncing a ball with two hands while counting down (dual tasking). Each training session lasted 60 min. The interventions were applied three times a week for 10 weeks. In this phase, faded feedback was provided allowing the patients to be dependent on their own visual and proprioceptor feedback from the trunk joints and musculature in addition to the vestibular system. Random type of practice was used in this phase to boost the long-term retention effects.

## 3. Statistical Analysis

The data have been analyzed using the SPSS software version 20 (SPSS Inc., Chicago, IL, USA). Descriptive statistics were calculated to summarize the demographic data of the participants. These demographic data were compared between groups using the *t*-test (*P* < 0.05). The outcome measure including TIS, PASS, FRT, and trunk ROMs was compared between and within groups using the *t*-test with level of significance set at *P* < 0.05 (paired *t*-test for within-group comparison and the independent *t*-test for between-group comparison).

## 4. Results

A total of 17 participants from the study group (task-specific training over a 10-week period) and 17 participants from the control group (only conventional rehabilitation program) were included in the analysis. Figure 1 shows the flow diagram for the study. No adverse effects of either intervention came across. Characteristics of both groups are presented in Table 1. To ensure the matching between groups, participants' characteristics were compared with regard to the following variables: age, sex, height, weight, duration of illness, and type of stroke. No significant differences were found between the 2 groups in terms of the demographic variables (*P* > 0.05).

**Figure 4:**
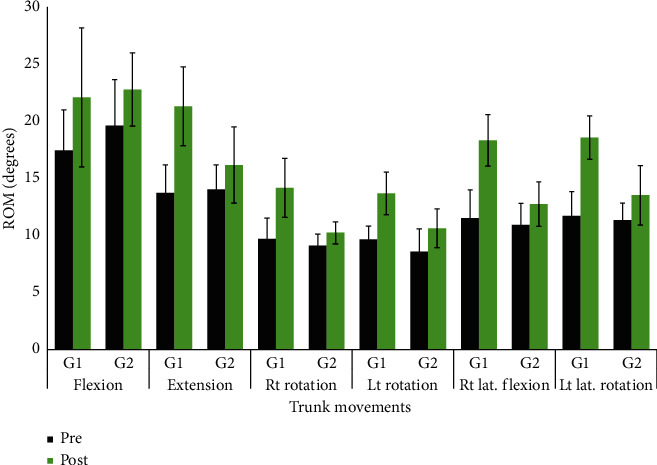
Within and in-between group comparison of the mean ± standard deviations (SD) of pre- and posttreatment measures of the trunk range of motions.

Table 2 presents the descriptive statistics, mean and standard deviation (SD) of dependent variables, and comparison within and between the two groups. No significant differences were found between the 2 groups in all outcome measures at the baseline assessment (*P* > 0.05). Both groups showed significant improvements in outcome measures including TIS, PASS, FRT, and trunk ROM during the 4 weeks between pre- and posttreatment assessment (*P* < 0.001). Patients in the study group improved significantly better when compared to the control group (*P* < 0.001). The results also reflect a significant difference among participants representing the study group when compared to the control group in terms of follow-up assessment (*P* < 0.05) (Figure 4).

## 5. Discussion

The aim of this study was to evaluate the effect of task-specific training on trunk performance after stroke. We used an examiner-blinded randomized controlled trial to compare the results of *G*_1_ receiving task-specific training to the results of the *G*_2_, which received conventional therapy based on neurodevelopmental therapy aiming at improving trunk control. Our results suggest that task-specific training resulted in significant improvement above what was found by conventional therapy on the TIS, PASS, and functional reach test. Secondary outcome measures including trunk ROM also showed significant improvement among patients in the study group when compared to those of the control group.

Improvements observed among patients representing the study and control groups can be attributed to the anatomical fact that the trunk muscles are bilaterally innervated and the fact that the axial muscles are rarely contracted unilaterally even when the arm produces a unilateral movement, to stabilize the trunk, which could enforce the contraction of the muscles on the paretic side by irradiation. This is consistent with Jean-Charles and colleagues who reported that proximal and axial muscles are innervated by inputs from both contralateral and ipsilateral corticospinal tracts [29]. Studies in animals have shown that the corticospinal tract sends collateral projections to nuclei from which reticulospinal neurons originate. These projections come from the areas of the motor cortex that control movements of proximal and axial muscles [30].

In the current study, patients representing the study group showed better progress than those in the control group. This could be attributed to the effect of using the principles of motor learning including task specificity, terminal feedback and the amount of time devoted to task repetition, giving the opportunity for more learning, in addition to dual-task training. The terminal feedback can improve the control of performed motor tasks. Knowledge of results is particularly useful in the earlier stages (phase I) because it can serve as a motivator and does not disturb the patient's attention during task performance. This is consistent with the other studies which concluded that feedback about performance is one of the most powerful variables affecting the learning of motor skills [31, 32]. In addition, Winstein and colleagues reported that terminal feedback which does not overload the patients during task performance is very helpful for retention [33].

It is well known that the best way to improve learning of new skills is to practice, practice, and more practice. The more time an individual spends practicing a new task, the more the opportunity to master that task [34]. This is consistent with studies which concluded that intense organized practice leads to improvements in functional performance [35], kinematics, and kinetics of movement with structural changes in the neurosubstrates of the brain which is parallel to improved movement competences [36–38].

Utilization of the dual-task training towards the end of the treatment (phase II) could help to improve automaticity of movement and switching task performance to the unconscious level. This is consistent with previous studies which reported that dual-task training plays an important role in improving the postural control that assists in improving balance abilities [39]. Another study proposed that merging motor and cognitive tasks as dual-task training brings a significant improvement on balance and daily living abilities among patients with stroke [40].

Our study had few limitations despite its strength. The study had a small sample size and did not assess the training effect at follow-up; assessing the training effect at follow-up might establish the long-term effect of the training interventions and ensure the retention phase of the motor learning. Future studies including larger sample size and modern technology in rehabilitation or virtual reality are recommended.

## Figures and Tables

**Table 1:** Subjects' characteristics.

	Task-specific training group, *G*_1_: *n* = 17	Conventional training group, *G*_2_: *n* = 17	*P*
Age (years)	58.76 ± 3.51	56.71 ± 3.74	0.108
Sex (males/females)	11/6	9/8	0.501
Weight (kg)	76.53 ± 5.91	75.76 ± 7.59	0.745
Height (cm)	169.53 ± 8.14	167.88 ± 7.79	0.551
Duration of illness (days)	27.82 ± 3.71	28.76 ± 3.29	0.440
Stroke (Hge/Inf)	3/14	4/13	0.862

Values are presented as mean ± SD and percentages. *P* is significant at *P* < 0.05.

**Table 2:** Within and between group comparison of the mean values of the measured trunk range of motions in degrees.

	Within groups						Between groups	
	Group 1		*P*	Group 2		*P*	Before	After
	Before	After		Before	After		*P*	*P*
TIS	17.47 ± 1.46	22.12 ± 0.99	<0.001	16.76 ± 1.52	18.47 ± 2.62	0.029	0.178	<0.001
PASS	20.53 ± 1.84	26.71 ± 2.85	**<0.001**	20.29 ± 2.05	22.06 ± 2.05	**0.018**	0.727	**<0.001**
FRT	11.56 ± 2.50	18.44 ± 3.14	**<0.001**	11.38 ± 3.36	14.13 ± 3.30	**0.027**	0.859	**0.001**
Flexion	19.18 ± 3.56	43.12 ± 6.10	**<0.001**	19.65 ± 4.03	22.82 ± 3.21	**0.016**	0.721	**<0.001**
Extension	13.76 ± 2.44	21.35 ± 3.46	**<0.001**	14.06 ± 2.16	16.18 ± 3.34	**0.037**	0.712	**<0.001**
*R* rotation	9.71 ± 1.83	14.18 ± 2.60	**<0.001**	9.118 ± 0.99	10.24 ± 0.97	**0.012**	0.062	**<0.001**
*L* rotation	9.65 ± 1.17	13.71 ± 1.86	**<0.001**	8.59 ± 2.00	10.63 ± 1.71	**0.021**	0.071	**<0.001**
*R* lateral flexion	11.53 ± 2.48	18.35 ± 2.26	**<0.001**	10.94 ± 1.89	12.76 ± 1.95	**0.009**	0.442	**<0.001**
*L* lateral flexion	11.71 ± 2.14	18.59 ± 1.91	**<0.001**	11.35 ± 1.50	13.53 ± 2.60	**0.006**	0.582	**<0.001**

Data are presented as mean ± standard deviations (SD). TIS: trunk impairment scale; PASS: postural assessment scale; FRT: functional reach test; R: right; L: left. *P* is significant at *P* < 0.05.

## Data Availability

The data (outcome measures raw data and photos) used to support the findings of this study are available from the corresponding author upon request.

## References

[1] Verheyden G., Nieuwboer A., De Wit L. (2008). Time course of trunk, arm, leg, and functional recovery after ischemic stroke. *Neurorehabilitation and Neural Repair*.

[2] Perlmutter S., Lin F., Makhsous M. (2010). Quantitative analysis of static sitting posture in chronic stroke. *Gait and Posture*.

[3] Ryerson S., Byl N. N., Brown D. A., Wong R. A., Hidler J. M. (2008). Altered trunk position sense and its relation to balance functions in people post-stroke. *Journal of Neurologic Physical Therapy*.

[4] Chern J.-S., Lo C.-Y., Wu C.-Y., Chen C.-L., Yang S., Tang F.-T. (2010). Dynamic postural control during trunk bending and reaching in healthy adults and stroke patients. *American Journal of Physical Medicine and Rehabilitation*.

[5] Verheyden G., Nieuwboer A., Feys H., Thijs V., Vaes K., De Weerdt W. (2005). Discriminant ability of the Trunk Impairment Scale: a comparison between stroke patients and healthy individuals. *Disability and Rehabilitation*.

[6] Titus A. W., Hillier S., Louw Q. A., Inglis-Jassiem G. (2018). An analysis of trunk kinematics and gait parameters in people with stroke. *African Journal of Disability*.

[7] Dickstein R., Shefi S., Marcovitz E., Villa Y. (2004). Anticipatory postural adjustment in selected trunk muscles in poststroke hemiparetic patients. *Archives of Physical Medicine and Rehabilitation*.

[8] Shin J. W., Don Kim K. (2016). The effect of enhanced trunk control on balance and falls through bilateral upper extremity exercises among chronic stroke patients in a standing position. *Journal of Physical Therapy Science*.

[9] Patel P. J., Bhatt T. (2018). Fall risk during opposing stance perturbations among healthy adults and chronic stroke survivors. *Experimental Brain Research*.

[10] Lee D.-K., Kim S.-H. (2018). The effect of respiratory exercise on trunk control, pulmonary function, and trunk muscle activity in chronic stroke patients. *Journal of Physical Therapy Science*.

[11] Karthikbabu S., Chakrapani M., Ganeshan S., Rakshith K. C., Nafeez S., Prem V. (2012). A review on assessment and treatment of the trunk in stroke: a need or luxury. *Neural Regeneration Research*.

[12] Karthikbabu S., Solomon J. M., Manikandan N., Rao B. K., Chakrapani M., Nayak A. (2011). Role of trunk rehabilitation on trunk control, balance and gait in patients with chronic stroke: a pre-post design. *Neuroscience and Medicine*.

[13] Hsieh C.-L., Sheu C.-F., Hsueh I.-P., Wang C.-H. (2002). Trunk control as an early predictor of comprehensive activities of daily living function in stroke patients. *Stroke*.

[14] Verheyden G., Nieuwboer A., De Wit L. (2007). Trunk performance after stroke: an eye catching predictor of functional outcome. *Journal of Neurology, Neurosurgery, and Psychiatry*.

[15] Jung S., Lee K., Kim M., Song C. (2017). Audiovisual biofeedback-based trunk stabilization training using a pressure biofeedback system in stroke patients: a randomized, single-blinded study. *Stroke research and treatment*.

[16] Mudie M. H., Winzeler-Mercay U., Radwan S., Lee L. (2002). Training symmetry of weight distribution after stroke: a randomized controlled pilot study comparing task-related reach, Bo bath and feedback training approaches. *Clinical Rehabilitation*.

[17] Pollock A. S., Durward B. R., Rowe P. J., Paul J. P. (2002). The effect of independent practice of motor tasks by stroke patients: a pilot randomized controlled trial. *Clinical Rehabilitation*.

[18] Dean C. M., Channon E. F., Hall J. M. (2007). Sitting training early after stroke improves sitting ability and quality and carries over to standing up but not to walking: a randomised controlled trial. *Australian Journal of Physiotherapy*.

[19] Thielman G. T., Dean C. M., Gentile A. M. (2004). Rehabilitation of reaching after stroke: task-related training versus progressive resistive exercise11No commercial party having a direct interest in the results of the research supporting this article has or will confer a benefit on the author(s) or on any organization with which the author(s) is/are associated. *Archives of Physical Medicine and Rehabilitation*.

[20] Salbach N. M., Mayo N. E., Robichaud-Ekstrand S., Hanley J. A., Richards C. L., Wood-Dauphinee S. (2005). The effect of a task-oriented walking intervention on improving balance self-efficacy poststroke: a randomized, controlled trial. *Journal of the American Geriatrics Society*.

[21] Arya K. N., Verma R., Garg R. K., Sharma V. P., Agarwal M., Aggarwal G. G. (2012). Meaningful task-specific training (MTST) for stroke rehabilitation: a randomized controlled trial. *Topics in Stroke Rehabilitation*.

[22] French B., Thomas L. H., Leathley M. J. (2007). Repetitive task training for improving functional ability after stroke. *The Cochrane Database of Systematic Reviews*.

[23] Thant A. A., Wanpen S., Nualnetr N. (2019). Effects of task-oriented training on upper extremity functional performance in patients with sub-acute stroke: a randomized controlled trial. *Journal of Physical Therapy Science*.

[24] Dang M., Ramsaran K. D., Street M. E. (2011). Estimating the accuracy of the Chedoke-McMaster stroke assessment predictive equations for stroke rehabilitation. *Physiotherapy Canada*.

[25] Verheyden G., Vereeck L., Truijen S. (2006). Trunk performance after stroke and the relationship with balance, gait and functional ability. *Clinical Rehabilitation*.

[26] Huang Y., Wang W., Liou T., Liao C., Lin L., Huang S. (2016). Postural Assessment Scale for Stroke Patients Scores as a predictor of stroke patient ambulation at discharge from the rehabilitation ward. *Journal of Rehabilitation Medicine*.

[27] Bennie S., Bruner K., Dizon A., Fritz H., Goodman B., Peterson S. (2003). Measurements of balance: comparison of the timed “up and go” test and functional reach test with the berg balance scale. *Journal of Physical Therapy Science*.

[28] Correll S., Field J., Hutchinson H., Mickevicius G., Fitzsimmons A., Smoot B. (2018). Reliability and validity of the halo digital goniometer for shoulder range of motion in healthy subjects. *International Journal of Sports Physical Therapy*.

[29] Jean-Charles L., Nepveu J.-F., Deffeyes J. E., Elgbeili G., Dancause N., Barthélemy D. (2017). Interhemispheric interactions between trunk muscle representations of the primary motor cortex. *Journal of Neurophysiology*.

[30] Davidson A. G., Buford J. A. (2004). Motor outputs from the primate reticular formation to shoulder muscles as revealed by stimulus-triggered averaging. *Journal of Neurophysiology*.

[31] Sidaway B., Ahn S., Boldeau P., Griffin S., Noyes B., Pelletier K. (2008). A comparison of manual guidance and knowledge of results in the learning of a weight-bearing skill. *Journal of Neurologic Physical Therapy*.

[32] Ishikura T. (2005). Average KR schedule in learning of timing: influence of length for summary knowledge of results and task complexity. *Perceptual and Motor Skills*.

[33] Winstein C. J., Pohl P. S., Lewthwaite R. (1994). Effects of physical guidance and knowledge of results on motor learning: support for the guidance hypothesis. *Research Quarterly for Exercise and Sport*.

[34] Schneiberg S., Sveistrup H., McFadyen B., McKinley P., Levin M. F. (2002). The development of coordination for reach-to-grasp movements in children. *Experimental Brain Research*.

[35] Kitago T., Liang J., Huang V. S. (2013). Improvement after constraint-induced movement therapy. *Neurorehabilitation and Neural Repair*.

[36] Laible M., Grieshammer S., Seidel G., Rijntjes M., Weiller C., Hamzei F. (2012). Association of activity changes in the primary sensory cortex with successful motor rehabilitation of the hand following stroke. *Neurorehabilitation and Neural Repair*.

[37] Treger I., Aidinof L., Lehrer H., Kalichman L. (2012). Constraint-induced movement therapy alters cerebral blood flow in subacute post-stroke patients. *American Journal of Physical Medicine and Rehabilitation*.

[38] Taub E., Uswatte G., Bowman M. H. (2013). Constraint-induced movement therapy combined with conventional neurorehabilitation techniques in chronic stroke patients with plegic hands: a case series. *Archives of Physical Medicine and Rehabilitation*.

[39] Silsupadol P., Shumway-Cook A., Lugade V. (2009). Effects of single-task versus dual-task training on balance performance in older adults: a double-blind, randomized controlled trial. *Archives of Physical Medicine and Rehabilitation*.

[40] Choi J. H., Kim B. R., Han E. Y., Kim S. M. (2015). The effect of dual-task training on balance and cognition in patients with subacute post-stroke. *Annals of Rehabilitation Medicine*.

